# A knowledge graph of clinical trials ($\mathop {\mathtt {CTKG}}\limits$)

**DOI:** 10.1038/s41598-022-08454-z

**Published:** 2022-03-18

**Authors:** Ziqi Chen, Bo Peng, Vassilis N. Ioannidis, Mufei Li, George Karypis, Xia Ning

**Affiliations:** 1 The Ohio State University, Columbus, USA; 2 Amazon Web Services AI, Palo Alto, USA; 3 Amazon Web Services Shanghai AI Lab, Shanghai, China

**Keywords:** Drug discovery, Drug development, Clinical trial design

## Abstract

Effective and successful clinical trials are essential in developing new drugs and advancing new treatments. However, clinical trials are very expensive and easy to fail. The high cost and low success rate of clinical trials motivate research on inferring knowledge from existing clinical trials in innovative ways for designing future clinical trials. In this manuscript, we present our efforts on constructing the first publicly available Clinical Trials Knowledge Graph, denoted as $\mathop {\mathtt {CTKG}}\limits$. $\mathop {\mathtt {CTKG}}\limits$ includes nodes representing medical entities in clinical trials (e.g., studies, drugs and conditions), and edges representing the relations among these entities (e.g., drugs used in studies). Our embedding analysis demonstrates the potential utilities of $\mathop {\mathtt {CTKG}}\limits$ in various applications such as drug repurposing and similarity search, among others.

## Introduction

**Figure 1:**
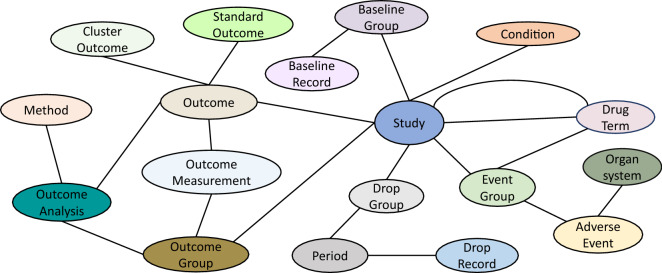
Schema of $$\mathop {\mathtt {CTKG}}\limits$$.

Clinical trials are studies aiming at determining the safety and efficacy of interventions, treatments or investigational drugs on human subjects^1^. Effective and successful clinical trials are essential in developing new drugs and advancing new treatments^2^. However, clinical trials are very expensive. As reported in Sertkaya et al.^3^, the average cost of a single phase in clinical trials ranges from 1.4 million up to 52.9 million US dollars. In addition, the success rate of the clinical trials is considerably low. As reported in Wong et al.^4^, for certain therapeutic groups like Oncology, the overall success rate of clinical trials could be as low as 3.4%. The high cost and low success rate of clinical trials motivate deliberate analysis of existing clinical trials, inferring knowledge from them, utilizing existing clinical trials in innovative ways, and accordingly carefully designing future clinical trials. The Access to Aggregate Content of ClinicalTrials.gov (AACT) database^5^ represents an effort in enhancing the accessibility and analysis of the clinical trial data. However, as a relational database, AACT is not formatted for the purpose of inferring new knowledge from existing clinical trials^6^. A Knowledge Graph (KG), instead, is a graph representation in which information entities are represented as nodes, and their relations are coded as edges connecting the corresponding nodes. In contrast to relational databases, KG has been proven^7–10^ to be an effective representation for knowledge inference purposes. Constructing a KG over clinical trial data is vital for advancing the analysis and research of clinical trials. In this manuscript, we present our work on constructing a such KG, referred to as Clinical Trials Knowledge Graph, denoted as $\mathop {\mathtt {CTKG}}\limits$, and also release $\mathop {\mathtt {CTKG}}\limits$ to the research community to facilitate advanced research using clinical trial data. $\mathop {\mathtt {CTKG}}\limits$ includes nodes representing medical entities (e.g., studies, drugs and conditions), and edges representing relations among these entities (e.g., drugs used in studies). Different from the recently released knowledge base^11^ that focuses only on extracting medical entities from the eligibility criteria in clinical trials, $\mathop {\mathtt {CTKG}}\limits$ includes more medical entities (e.g., adverse events and outcomes) and also the relations among these entities. The rich information in $\mathop {\mathtt {CTKG}}\limits$ could enable more biomedical applications (e.g., adverse drug event prediction, outcome prediction) than the existing knowledge base in clinical trials. Figure 1 presents the schema of $\mathop {\mathtt {CTKG}}\limits$. The detailed descriptions of nodes and edges in $\mathop {\mathtt {CTKG}}\limits$ will be presented in “Nodes in CTKG” section. To the best of our knowledge, $\mathop {\mathtt {CTKG}}\limits$ is the first publicly available clinical trials knowledge graph in the scientific research community. The results of the embedding analysis over $\mathop {\mathtt {CTKG}}\limits$ demonstrate its potential utilities in various applications such as drug repurposing and similarity search, among others.

## Results

### $\mathop {\mathtt {CTKG}}\limits$ schema

Figure 1 presents the schema of $\mathop {\mathtt {CTKG}}\limits$. The schema presents the different information entities involved in clinical trials, represented as nodes, and the relations among them, represented as edges. There are 18 types of nodes and 21 types of edges in $\mathop {\mathtt {CTKG}}\limits$. Each node and edge type has attributes describing the properties of the nodes and edges. The statistics of different nodes and edges are presented in Tables 1 and 2, respectively. Detailed descriptions of node and edge attributes are available in the online documentation of $\mathop {\mathtt {CTKG}}\limits$^12^. We developed a web portal (https://u.osu.edu/ning.104/data-set/ctkg/ctkg-webportal/) for users to visualize $\mathop {\mathtt {CTKG}}\limits$ and access its nodes and edges interactively.

**Table 1:** Statistics of node types in $\mathop {\mathtt {CTKG}}\limits$.

Node type	Is study specific?	Statistics
Study	Yes	8210
Condition	No	1394
Drug term	No	2548
Event group	Yes	22,725
Adverse event	No	18,546
Organ	No	27
Baseline group	Yes	27,068
Baseline record	Yes	315,533
Drop group	Yes	22,272
Period	Yes	34,330
Drop record	Yes	123,627
Outcome group	Yes	32,499
Method	No	907
Outcome measurement	Yes	690,626
Outcome analysis	Yes	107,294
Outcome	Yes	88,386
Standard outcome	No	492
Cluster outcome	No	200

**Table 2:** Statistics of relation types in $\mathop {\mathtt {CTKG}}\limits$.

Relation type	Node type 1	#Node 1	Node type 2	#Node 2	#Relations
Study-Condition	Study	8210	Condition	1394	17,259
Study-EventGroup	Study	8172	Event group	22,725	22,725
Study-BaselineGroup	Study	8209	Baseline group	27,068	27,068
Study-DropGroup	Study	8210	Drop group	22,272	22,272
Study-OutcomeGroup	Study	8210	Outcome group	32,499	32,499
Study-Outcome	Study	8210	Outcome	88,386	88,386
Study-StudiedDrug	Study	8169	Drug term	2373	20,982
Study-UsedDrug	Study	2234	Drug term	920	3992
Drug-EventGroup	Drug term	2201	Event group	21,790	31,528
EventGroup-AdverseEvent	Event group	20,571	Adverse event	18,546	966,450
AdverseEvent-Organ	Adverse event	18,546	Organ	27	18,546
BaselineGroup-BaselineRecord	Baseline group	27,068	Baseline record	315,533	315,533
DropGroup-Period	Drop group	22,272	Period	34,330	34,330
Period-DropRecord	Period	25,956	Drop record	123,627	123,627
OutcomeGroup-OutcomeMeasurement	Outcome group	32,240	Outcome measurement	690,541	690,541
OutcomeGroup-OutcomeAnalysis	Outcome group	23,923	Outcome analysis	107,294	209,314
OutcomeAnalysis-Method	Outcome analysis	91,463	Method	907	91,463
Outcome-OutcomeAnalysis	Outcome	45,689	Outcome analysis	107,294	107,294
Outcome-OutcomeMeasurement	Outcome	85,905	Outcome measurement	690,626	690,626
Outcome-ClusterOutcome	Outcome	88,244	Cluster outcome	200	88,244
Outcome-StandardOutcome	Outcome	50,342	Standard outcome	492	58,819

Columns represent: “Relation type”: the type of relation; “Node type 1”: the type of head nodes in the relations; “#Node 1”: the number of unique head nodes with the relations; “Node type 2”: the type of tail nodes in the relations; “#Node 2”: the number of unique tail nodes with the relations; “#Relations”: the number of relations of a relation type.

### Nodes in $\mathop {\mathtt {CTKG}}\limits$

Each *study* node represents a clinical trial and is associated with the primary properties of that clinical trial as node attributes. The properties of each *study* node describe the purposes, phases and the protocols of the corresponding clinical trial. Each *study* node links to *condition* nodes, *drug* nodes, *outcome* nodes and multiple types of *group* nodes via one-to-many relationships. Each *condition* node describes a disease or syndrome that is extracted from the AACT and studied by some clinical trials.

Each *drug-term* node represents the drug used in clinical trials, and is identified by the extracted drug mention (“Drug mentions and normalization” section). The *drug-term* nodes connect with study nodes via *StudiedDrug* and *UsedDrug* relations. The *StudiedDrug* relation connects studies and drug terms that are studied in at least one study group of the corresponding clinical trial, and the *UsedDrug* relation connects studies and the auxiliary drug terms such as pain reducers. Please refer to “Drug mentions and normalization” section for more details.

Each *outcome* node represents an outcome measure used to evaluate the efficacy of interventions in the clinical trials, and has the name and the description of the outcome measure as attributes. For example, the study “NCT04322526” uses the outcome “changes in blood oxygenation level-dependent (BOLD)” to evaluate the efficacy of interventions. Each *outcome* node is connected to a *study* node, representing that this specific outcome is used within the study. Note that unlike the *condition* node linking to multiple *study* nodes, each *outcome* node links to a unique *study* node. This is due to the complexity and the diversity of outcome measures, which makes it difficult to be shared across multiple *study* nodes. Each *outcome* node also links to one *cluster-outcome* node and multiple *standard-outcome* nodes. The connection between the *outcome* node and the *cluster-outcome* node represents that the name of the outcome can be assigned to the cluster represented by the *cluster-outcome* node, while the connection between the *outcome* node and the *standard-outcome* node represents that the name or the description of the outcome contains the standard outcome measure. Please refer to “Outcome extraction and outcome clustering” section for more details.

Each *group* node represents a study arm or a comparison group, that is, a group of participants who receive a specific intervention. There are multiple types of *group* nodes as follows:

- *event-group* node. The information described by each *event-group* node is the number of participants within the group affected by specific types of adverse events. Each *event-group* node is connected to multiple *drug* nodes representing the drugs used in the event group, and *adverse-event* nodes representing the specific adverse events that occurred in the event group. Each *adverse-event* node also links to an *organ-system* node representing the affected organ system.
- *baseline-group* node. Each *baseline-group* node represents a group of participants with their demographic attributes (e.g., “Age” and “Ethnicity”) or study-specific attributes (e.g., “Baseline Modified Gingival Index”). Each *baseline-group* node is connected to one or multiple *baseline-record* nodes.
- *drop-group* node. Each *drop-group* node represents a group of participants with their withdrawal information. Each *drop-group* node is connected to one or multiple *period* nodes. Each *period* node represents an interval of the study (e.g., “First Intervention” and “Part 1: Treatment Period 1”), and has attributes describing the number of participants at the beginning and the end of the period. Each *period* node can link to multiple *drop-record* nodes. Each *drop-record* node includes a withdrawal reason and documents the number of the participants in the group withdrawing with this reason in a period.
- *outcome-group* node. An *outcome-group* node has the information on the efficacy of the studied interventions on the participants. The efficacy is evaluated by different outcome measures and analyzed by different statistical test methods with the measurements. For example, an *outcome-group* node could represent a group of 17 participants receiving Naltrexone as the intervention, and the efficacy of Naltrexone was evaluated using the results from this group of participants; these 17 participants and the efficacy evaluation are included in an *outcome-group* node. Other nodes related to the efficacy measures of interventions are as below:
  - *method* node. Each *method* node represents a statistical hypothesis testing method that is used to make inference or draw conclusion statistically from the data collected in clinical trials. For example, the variance analysis method “ANOVA” could be a *method* node that is used to test the superiority of an intervention compared with a control in a clinical trial. Each *method* node is connected to multiple *outcome-analysis* nodes, representing that the method is used to conduct the analyses. Please refer to “Statistical analysis method normalization” section for more details about the normalization of method names.
  - *outcome-measurement* node. Each *outcome-measurement* node represents the measurement of a specific outcome measure (i.e., *outcome* node) on the corresponding group of participants. For example, the measurement of the average changes in BOLD (i.e., *outcome* node) on the 17 participants in the *outcome-group* node is 1.23 with a standard deviation 1.07, and is represented as a *outcome-measurement* node. Each *outcome-measurement* node links to one *outcome* node and one *outcome-group* node.
  - *outcome-analysis* node. Each *outcome-analysis* node represents a statistical analysis on a specific outcome measure by comparing multiple outcome groups using a statistical testing method. For example, the analysis of the outcome “average changes in BOLD” via the statistical testing method “paired t-test” on two groups of participants receiving the intervention “Naltrexone” and placebo, is represented as an *outcome-analysis* node; the *p*-value of the analysis is 0.002, indicating that the alternative “Naltrexone will block contextual processing” can be accepted at the significance level of 0.005. Each *outcome-analysis* node links to one *outcome* node, one *method* node and multiple *outcome-group* nodes.

Note that the different types of *group* nodes for a study could represent the same participant group with different information. According to AACT, using a single group to uniquely represent a participant group in the study is impossible due to the complicated designs of clinical trials. Therefore, we followed AACT and used different types of *group* nodes to represent different types of information of the clinical trials.

## Embedding analysis

We conducted an embedding analysis to evaluate the quality of $\mathop {\mathtt {CTKG}}\limits$ and demonstrate its utilities in various important applications. We applied TransE ^13^, a state-of-the-art graph embedding method, to generate embeddings for nodes in $\mathop {\mathtt {CTKG}}\limits$. These computable embeddings can benefit various downstream tasks. For example, we could establish similarities among nodes using their embeddings. The similarities enable fast retrieval of nodes corresponding to similar medical entities and could facilitate applications such as drug repurposing and similar study search, as will be discussed below. Note that TransE generates node embeddings based on the topology of the graph (i.e., node attributes are not considered). We used the TransE implementation in DGL-KE ^14^, which is a high-performance python library on top of the Deep Graph Library (DGL ^15^). DGL is a python library for deep learning on graphs and enables training models on large-scale graphs in a convenient way. Based on DGL, DGL-KE provides many popular KG embedding algorithms like TransE for users to learn KG embeddings that can be used for many different applications ^9^. Other KG embedding methods are also applicable for the following analyses. Detailed information on KG embedding methods is available in a survey ^16^.

### $\mathop {\mathtt {CTKG}}\limits$ for drug repurposing

In this analysis, we evaluated the utilities of $\mathop {\mathtt {CTKG}}\limits$ for drug repurposing—a strategy to identify new therapeutic indications for existing drugs ^17^. Particularly, we assessed if the high similarities between the *condition* node embeddings and *drug-term* node embeddings indicate the high potential of the corresponding drugs in treating the conditions. For the evaluation, we calculated the cosine similarities between all the *condition* nodes and *drug-term* nodes, and identified the top-10 most similar pairs. Among these 10 pairs, we found that 5 of them have evidence demonstrated by the literature indicating potential repurposability, as presented in Table 3. For example, the *condition* node “Diabetes Mellitus, Type 2” has a similarity 0.597 with the *drug-term* node “Benzoates”; as demonstrated in the literature ^18^, Alogliptin Benzoates, an agent of Benzoates, is now available for the treatment of Type 2 Diabetes. Similarly, the *condition* node “Lung Neoplasms” has a similarity 0.574 with the *drug-term* node “Triterpenes”, and as demonstrated in the literature ^19^, Triterpenes have anti-cancer properties against Lung Neoplasms. Please note the average similarity between *condition* nodes and *drug-term* nodes is -0.032, and thus the above similarities are significantly high. In addition, the above drugs are not studied for their highly-similar conditions in any $\mathop {\mathtt {CTKG}}\limits$ studies (i.e., no existing edges connecting the *condition* nodes and *drug-term* nodes). Thus, the above results demonstrate the utilities of $\mathop {\mathtt {CTKG}}\limits$ for drug repurposing. Other similar *condition* and *drug-term* node pairs, for example, “Squamous Cell Carcinoma of Head and Neck” and “Naloxone” with similarity 0.565, and “Lung Neoplasms” and “Uric Acid” with similarity 0.564, may enable new hypothesis generation for innovative investigation and findings.

**Table 3:** Similar *condition* nodes and *drug-term* nodes for drug repurposing.

Similarity	Similar nodes	Possible evidence
0.597	Diabetes Mellitus, Type 2	Alogliptin benzoate, an agent of Benzoates, is now available for treatment of type 2 diabetes^18^
	Benzoates	
0.587	Diabetes Mellitus, Type 2	Pulmonary surfactant involves in delaying the fetal lung biochemical maturation by maternal diabetes^38^
	Pulmonary Surfactants	
0.576	Diabetes Mellitus	Pulmonary surfactant involves in delaying the fetal lung biochemical maturation by maternal diabetes^38^
	Pulmonary Surfactants	
0.574	Lung Neoplasms	Representatives of triterpenes show anti-cancer properties against multiple types of cancer including lung cancer^19^
	Triterpenes	
0.562	Lung Neoplasms	Pregnenediones shows promising activity against lung cancer cell lines^39^
	Pregnenediones	

The average cosine similarity between *condition* nodes and *drug-term* nodes is - 0.032.

### $\mathop {\mathtt {CTKG}}\limits$ for similar medical entity retrieval

In this analysis, we evaluated whether $\mathop {\mathtt {CTKG}}\limits$ enables high-quality node embeddings for similar medical entity retrieval tasks. Particularly, we focused on the retrieval of similar studies, and the retrieval of similar conditions, drugs, adverse events and outcomes. All these retrieval tasks are common and useful in designing new clinical trials ^20^.

#### Similar study retrieval

$\mathop {\mathtt {CTKG}}\limits$ can support the search and retrieval of similar studies. To demonstrate this, we first identified the top-5 most similar pairs of *study* nodes using cosine similarity over their embeddings. In each identified pair, we randomly selected one *study* node, and identified its top-5 most similar *study* nodes. Table 4 presents the selected *study* nodes and their top-5 most similar *study* nodes. As presented in Table 4, the identified similar studies all investigated similar drugs or conditions. For example, study “NCT00795769” and its top-5 most similar studies investigated the prevention of the side effects caused by the stem cell transplant, or conditions that could be treated by the stem cell transplant; study “NCT01431274” and its top-5 most similar studies all investigated the therapies for the Chronic Obstructive Pulmonary Disease (COPD). These results show the utilities of $\mathop {\mathtt {CTKG}}\limits$ for retrieving similar studies, which could facilitate new clinical trial design.

**Table 4:** Similar *study* nodes.

*Study* node	Similarity	Similar nodes	Possible evidence
NCT00795769	0.840	NCT01789255	NCT00918333 and NCT00720109 investigate therapies for conditions that could be treated by the stem cell transplant (e.g., Lymphoma). All the other studies are on preventing side effects following the stem cell transplant
	0.741	NCT00918333	
	0.713	NCT00105001	
	0.672	NCT00293384	
	0.629	NCT00720109	
NCT01431274	0.826	NCT01431287	All the studies investigate therapies for the Chronic Obstructive Pulmonary Disease (COPD)
	0.737	NCT01559116	
	0.721	NCT02796651	
	0.716	NCT00782509	
	0.709	NCT00931385	
NCT00137111	0.825	NCT00866307	All the studies investigate therapies for different sub-types of Leukemia (e.g., Acute Lymphoblastic Leukemia, Acute Myeloid Leukemia)
	0.748	NCT00720109	
	0.747	NCT00136084	
	0.744	NCT00808639	
	0.724	NCT00119262	
NCT00782509	0.784	NCT00796653	All the studies investigate the safety and efficacy of BI 1744 CL in patients with COPD
	0.749	NCT00793624	
	0.735	NCT01040793	
	0.724	NCT01040130	
	0.700	NCT00782210	
NCT02105688	0.801	NCT02252016	All the studies investigate therapies for the Chronic Hepatitis C Virus (HCV)
	0.688	NCT02105467	
	0.662	NCT02358044	
	0.655	NCT01544920	
	0.652	NCT02216422	

The average cosine similarity among *Study* nodes is 0.301.

#### Similar medical concept retrieval

$\mathop {\mathtt {CTKG}}\limits$ can also support the retrieval of other similar medical concepts. To demonstrate this, we identified the top-10 most similar pairs of *condition* nodes, *drug-term* nodes, *adverse-event* nodes, and *standard-outcome* nodes, as in Tables 5, 6, 7, and 8, respectively, using cosine similarities over their embeddings. As presented in Table 5, the identified similar *condition* nodes all share some commonalities. For example, *condition* node “Nephritis” and “Lupus Nephritis” have a similarity 0.997 (average *condition* similarity is 0.331), and Lupus Nephritis is a common sub-type of Nephritis. We also found a similar trend in Table 6, 7 and 8. For example, *durg-term* nodes “ABT-267” and “Macrocyclic Compound” have a similarity 0.997 (average *drug-term* similarity is 0.254), and both drugs could be used to treat Hepatitis C Virus (HCV) infection ^21,22^. In addition, the two drugs are studied together in multiple studies such as NCT01458535, NCT01464827 and NCT01563536. In Table 7 for *adverse-event* nodes, “Blood Luteinising Hormone” is very similar to “Uterus Myomatosus” (cosine similarity 0.995; average *adverse-event* similarity is 0.329). The high similarity could be due to the fact that Luteinising Hormone can affect the development and growth of Uterus Myomatosus by stimulating the production of estrogen ^23^. Note that Luteinising Hormone and Uterus Myomatosus are not present together in any of the $\mathop {\mathtt {CTKG}}\limits$ studies; therefore, such similar pairs demonstrate the effectiveness of $\mathop {\mathtt {CTKG}}\limits$ on retrieving similar/related adverse events. In Table 8 for *standard-outcome* nodes, “Aspartate Aminotransferase” is very similar to “Alanine Aminotransferase” in their embeddings (cosine similarity 0.986; average *standard-outcome* similarity is 0.315), and both measure the amount of two enzymes made by liver in the blood and can be tested to check the liver damage. These results demonstrate that $\mathop {\mathtt {CTKG}}\limits$ can facilitate the search and retrieval of medical entities in the context of clinical trials that carry similar/related information.

**Table 5:** Top-10 most similar *condition* nodes.

Similarity	Similar nodes	Possible evidence
0.997	Nephritis	Lupus Nephritis is a common sub-type of Nephritis
	Lupus Nephritis	
0.997	Hepatitis	Hepatitis A is a special sub-type of Hepatitis
	Hepatitis A	
0.996	Rhinitis	Rhinitis, Allergic is a sub-type of Rhinitis caused by allergy
	Rhinitis, Allergic	
0.996	Urinary Bladder Disease	Urinary Bladder Disease is a special sub-type of Urologic Disease
	Urologic Disease	
0.996	Arthritis	Arthritis, Rheumatoid is a chronic inflammatory Arthritis
	Arthritis, Rheumatoid	
0.995	Neovascularization, Pathologic	Both of the conditions are sub-types of Neovascularization
	Choroidal Neovascularization	
0.995	Diabetes Mellitus	Diabetes Mellitus, Type 2 is a common sub-type of Diabetes Mellitus
	Diabetes Mellitus, Type 2	
0.994	Alopecia	Alopecia Areata is a sub-type of Alopecia
	Alopecia Areata	
0.994	Depression	Depression is also known as major Depressive Disorder in Clinics^40^
	Depressive Disorder	
0.993	Keratosis	Keratosis, Actinic is a sub-type of Keratosis
	Keratosis, Actinic	

The average cosine similarity among *condition* nodes is 0.311.

**Table 6:** Top-10 most similar *drug-term* nodes.

Similarity	Similar nodes	Possible evidence
0.997	ABT-267	Both ABT-267 and Macrocyclic Compounds could be used to treat Hepatitis C Virus (HCV) infection^21,22^
	Macrocyclic Compounds	
0.996	Pulmonary Surfactants	Pulmonary Surfactants is a type of Surface-Active Agents^41^
	Surface-Active Agents	
0.995	Phenylethyl Alcohol	Phenylethyl Alcohol and LY2216684 are studied together in study NCT00922636, NCT01243957 and NCT01380691
	LY2216684	
0.994	Thioguanine	Thioguanine is a substitute of Mercaptopurine in treating childhood lymphoblastic leukaemia^42^
	Mercaptopurine	
0.993	Cilastatin	Cilastatin and Imipenem are commonly used together as a treatment for serious infections^43^
	Imipenem	
0.985	Metylperon	Metylperon is an atypical antipsychotic of the Butyrophenone chemical class^44^
	Butyrophenones	
0.983	Ubiquinone	Ubiquinone is a form of Coenzyme Q10^45^
	Coenzyme Q10	
0.982	PHiD-CV Vaccine	Both of the drug terms are vaccines for diphtheria^46,47^
	VAXELIS	
0.982	Propafenone	Both Propafenone and Sotalol could maintain sinus rhythm for patients with recurrent symptomatic atrial fibrillation^48^
	Sotalol	
0.980	SNAP25 Protein	SNAP25 Protein could block Acetylcholine from releasing at the neuromuscular junction^49^
	Acetylcholine	

The average cosine similarity among *drug-term* nodes is 0.254.

**Table 7:** Top-10 most similar *adverse-event* nodes.

Similarity	Similar nodes	Possible evidence
0.998	Blood Luteinising Hormone Increased	Luteinising Hormone (LH) can affect the growth of Uterus Myomatosus by controling the level of estrogen^23^
	Uterus Myomatosus	
0.997	Inpatient Hospitalization	Excess length of inpatient hospitalization can lead to ulceration^50^
	Ulceration	
0.997	Major Bleeding Event	Patients receiving hemodialysis are at risk for major bleeding event and catheter-related infection^51^
	Infection with Unknown Anc, Catheter-Related	
0.996	Blood Luteinising Hormone Increased	The level of LH is related to uterine bleeding^52^
	Major Bleeding Event	
0.995	Blood Luteinising Hormone Increased	LH may regulate skin functions via LH receptors on skin^53^
	Skin Procedural Complication	
0.995	Skin Procedural Complication	Both are similar to the *adverse-event* node “Blood Luteinising Hormone Increased”
	Uterus Myomatosus	
0.995	Infection with Unknown Anc, Catheter-Related	Patients with prostatic obstruction often receive urinary catheters, and are at risk for catheter-related infection^54^
	Prostatic Obstruction	
0.994	Gi Tract Perforation	Diabetes can induce Gi Tract Perforation^55^
	Latent Autoimmune Diabetes in Adults	
0.994	Cervix Carcinoma Stage III	Both of the adverse events are related with Uterus
	Vanishing Twin Syndrome	
0.994	Major Bleeding Event	Uterus Myomatosus can associate with major bleeding event^56^
	Uterus Myomatosus	

The average cosine similarity among *adverse-event* nodes is 0.329.

**Table 8:** Top-10 most similar *standard-outcome* nodes.

Similarity	Similar nodes	Possible evidence
0.986	Aspartate Aminotransferase	Both are enzymes that are tested to check liver damage^57^
	Alanine Aminotransferase	
0.955	Swollen Joint Count	Both are used to assess patients with rheumatoid arthritis^58^
	Tender Joint Count	
0.952	Calcium	Both are electrolyte that can be tested to monitor a range of medical conditions^59^
	Potassium	
0.952	Incomplete Response	Both are used to assess the response to treatment^60^
	Partial Response	
0.946	Aspartate Aminotransferase	Both can be tested to check liver damage^57,61^
	Blood Urea Nitrogen	
0.941	Potassium	Both are included in basic metabolic panel blood test^59^
	Blood Urea Nitrogen	
0.940	Calcium	Both are included in basic metabolic panel blood test^59^
	Blood Urea Nitrogen	
0.930	Alanine Aminotransferase	Both can be tested to check kidney damage^57,61^
	Blood Urea Nitrogen	
0.930	Hemoglobin A1c	Hemoglobin A1c represents the hemoglobin in the blood that has glucose attached to it^62^
	Hemoglobin	
0.923	Erythrocyte Sedimentation Rate	Disease Activity Score 28 can be calculated based on Erythrocyte Sedimentation Rate^63^
	Disease Activity Score 28	

The average cosine similarity among *standard-outcome* nodes is 0.315.

### $\mathop {\mathtt {CTKG}}\limits$ for other applications

$\mathop {\mathtt {CTKG}}\limits$ could also enable other potential applications such as adverse drug event prediction and outcome prediction, etc. Specifically, for the adverse drug event prediction, we could employ knowledge reasoning methods^24^ over $\mathop {\mathtt {CTKG}}\limits$, and infer new adverse events of drugs using the existing or predicted paths from *drug-term* nodes to *adverse-event* nodes in $\mathop {\mathtt {CTKG}}\limits$. For the outcome prediction, we could employ link prediction methods^9,13^ to infer new edges between *study* nodes and *outcome* nodes based on the existing ones in $\mathop {\mathtt {CTKG}}\limits$. Overall, $\mathop {\mathtt {CTKG}}\limits$ could facilitate new knowledge discovery and benefit the design of new clinical trials, and also improve the success rate of future clinical trials. We released the code for drug repurposing and similar node retrieval (“Data availability” section). For the link prediction applications, please refer to the examples in DGL ^25^ for a concrete implementation.

## Discussion

In this manuscript, we presented and released a new knowledge graph $\mathop {\mathtt {CTKG}}\limits$ for clinical trials. We also described our methods in generating $\mathop {\mathtt {CTKG}}\limits$. We demonstrated the potential utilities of $\mathop {\mathtt {CTKG}}\limits$ in drug repurposing and similarity search, among others, via embedding analysis over $\mathop {\mathtt {CTKG}}\limits$. Currently, $\mathop {\mathtt {CTKG}}\limits$ only includes studies that have both drug interventions and reported outcomes. However, incomplete studies (e.g., studies not started or without reported outcomes), and studies without drug interventions (e.g., studies for medical devices) could also contain valuable knowledge for the design of future clinical trials. Therefore, we will enrich $\mathop {\mathtt {CTKG}}\limits$ with more studies in the future research. In addition, current $\mathop {\mathtt {CTKG}}\limits$ does not contain all the important information for drug discovery and development. For example, $\mathop {\mathtt {CTKG}}\limits$ does not have the interactions between drugs/molecules and proteins/diseases, nor the interactions among proteins. Missing such information may limit the potential of $\mathop {\mathtt {CTKG}}\limits$ for a much wider range of applications (e.g., to predict if a new molecule for a disease can survive from clinical trials). In the future research, we will align $\mathop {\mathtt {CTKG}}\limits$ with other knowledge bases^10,26,27^ and integrate more and diverse information into $\mathop {\mathtt {CTKG}}\limits$ to enable more applications using $\mathop {\mathtt {CTKG}}\limits$. Moreover, $\mathop {\mathtt {CTKG}}\limits$ embeds rich textual information (e.g., title and description) and heterogeneous data types (e.g., numerical, categorical and textual data) as node attributes, which encourages a much borader spetrum of research (e.g., deep graph embedding, link prediction) and more complicated methods to be developed to leverage such information for better translational clinical trial design. We will also investigate attribute-sensitive KG embedding methods ^28^ to better leverage $\mathop {\mathtt {CTKG}}\limits$.

## Methods

**Figure 2:**
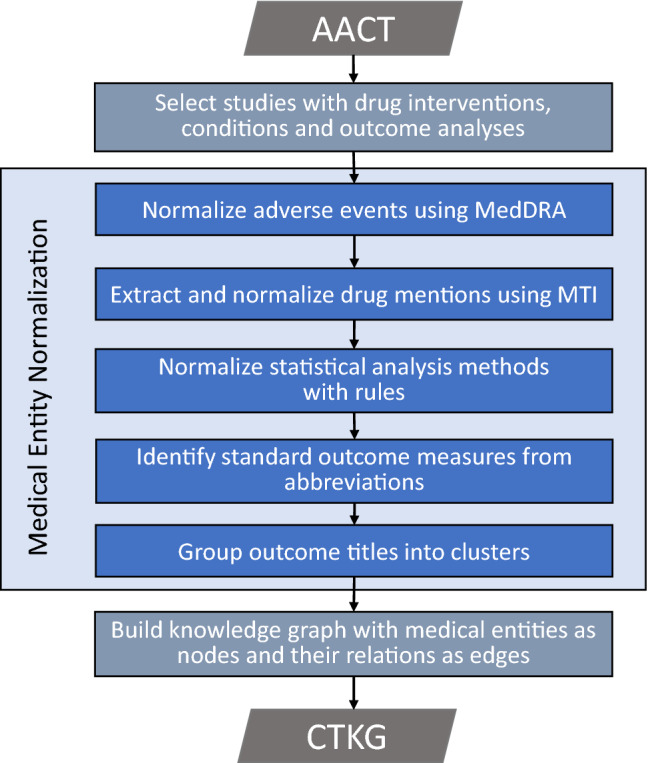
Flow chart of $\mathop {\mathtt {CTKG}}\limits$ construction.

$\mathop {\mathtt {CTKG}}\limits$ represents each medical entity (e.g., a clinical trial, also referred to as a study; a drug; an adverse event) as a single node. To develop $\mathop {\mathtt {CTKG}}\limits$, we extracted the medical entities from the Access to Aggregate Content of ClinicalTrials.gov (AACT) database ^5^. We then normalized multiple expressions of a same medical entity into a single one. Figure 2 presents the overview of development of $\mathop {\mathtt {CTKG}}\limits$.

### Clinical trials data

The clinical trials data in $\mathop {\mathtt {CTKG}}\limits$ is collected from the AACT database. AACT is a publicly available relational database, which contains the information of every clinical trial registered in ClinicalTrials.gov, and is updated on a daily basis. In AACT, each clinical trial, also referred to as a study, is associated with a unique National Clinical Trial (NCT) ID, and all the information of a clinical trial is stored in 45 different tables. For example, information representing the medications, procedures and other actions provided or conducted in a clinical trial is stored in two tables: “interventions” and “browse interventions”; information representing the measurements used to evaluate the safety and efficacy of drugs or procedures studied in clinical trials is stored in the table “outcomes.” All the tables and their schemas are publicly available ^29^. Until July 2020, 344,500 clinical trials have been registered in ClinicalTrials.gov and included in AACT. We selected all the studies that have drug interventions, conditions and outcome analyses into $\mathop {\mathtt {CTKG}}\limits$. Specifically, we excluded 232,274 studies that do not have drug interventions, and 103,047 studies that do not have outcome analyses. Among 9,179 remaining studies, we excluded 969 studies without the conditions, resulting 8210 studies in $\mathop {\mathtt {CTKG}}\limits$. Note that we did not consider clinical trials that are not on drug inventions, such as physical therapies, behavioral therapies or medical devices. We will update $\mathop {\mathtt {CTKG}}\limits$ with more studies in the future as new studies on drug interventions become available.

Note that $\mathop {\mathtt {CTKG}}\limits$ does not include all the tables in AACT. For example, $\mathop {\mathtt {CTKG}}\limits$ does not include tables such as “Sponsors”, “Overall officials” and “Result contacts” because they are not directly related to the design and results of clinical trials, and including them may not significantly benefit the knowledge graph in analyzing the relations among medical entities. Other AACT tables such as “Provided documents” and “Documents” contain the links to detailed study protocols, informed consent forms and statistical analysis plans, etc. These documentations have rich textual information that might be complementary to the structural relations represented by $\mathop {\mathtt {CTKG}}\limits$. However, such information is highly specific to each individual clinical trial, and does not help establish new relations across clinical trials if no natural language processing is applied first, which by itself is highly non-trivial. Therefore, $\mathop {\mathtt {CTKG}}\limits$ does not include such tables; instead, $\mathop {\mathtt {CTKG}}\limits$ uses AACT’s original study IDs so that all such information can still be retrieved from AACT if needed. $\mathop {\mathtt {CTKG}}\limits$ does not include other AACT tables such as “Calculated values”, “Design outcomes” and “Design group interventions” because information in such tables is already included in other tables that $\mathop {\mathtt {CTKG}}\limits$ includes. Table 9 summarizes the AACT tables that are included and are not included in $\mathop {\mathtt {CTKG}}\limits$.

**Table 9:** AACT tables included and not included in $\mathop {\mathtt {CTKG}}\limits$.

Included in $\mathop {\mathtt {CTKG}}\limits$	Not included in $\mathop {\mathtt {CTKG}}\limits$
Baseline counts	Brief summaries
Baseline measurements	Calculated values
Browse conditions	Central contacts
Browse intervention	Countries
Conditions	Design group interventions
Designs	Design groups
Drop withdrawals	Design outcomes
Eligibilities	Detailed descriptions
Interventions	Documents
Id information	Facilities
Milestones	Facilities contacts
Outcome measure	Facility investigator
Outcome analyses	Intervention other names
Outcome analysis groups	Ipd information types
Outcomes	Keywords
Participant flows	Links
Reported events	Overall officials
Result groups	Pending results
Studies	Provided documents
Study references	Responsible parties
	Result agreements
	Result contacts
	Sponsors

### Adverse event normalization

In AACT, we could find the adverse events ($\mathop {\mathtt {AE}}\limits$) , represented by AE terms, happened among the participants in the “reported events” table. Many $\mathop {\mathtt {AE}}\limits$ terms listed in the table could be mapped to the Medical Dictionary for Regulatory Activities ($\mathop {\mathtt {MedDRA}}\limits$ $\circledR$^30^). $\mathop {\mathtt {MedDRA}}\limits$ $\circledR$ is the international medical terminology developed under the auspices of the International Council for Harmonisation of Technical Requirements for Pharmaceuticals for Human Use (ICH). More specifically, we found 28,677 unique $\mathop {\mathtt {AE}}\limits$ terms in which 13,995 terms could be directly mapped to the $\mathop {\mathtt {MedDRA}}\limits$ dictionary. In $\mathop {\mathtt {CTKG}}\limits$, such terms are also referred to as $\mathop {\mathtt {MedDRA}}\limits$ terms. We normalized the remaining 14,682 $\mathop {\mathtt {AE}}\limits$ terms that are not in the $\mathop {\mathtt {MedDRA}}\limits$ $\circledR$ to $\mathop {\mathtt {MedDRA}}\limits$ terms as follows:

- We removed parenthesized contents (e.g., “Altered pitch perception (pitch seemed lower)”). The contents in parentheses are typically explanations or afterthoughts so removing them would not significantly affect the major meanings.
- We removed words or phrases that specify the auxiliary information (e.g., “left”, “right”, “Baseline Phase”) or the time frame (e.g., “for 12 hours”) of adverse events. We observed that these words or phrases are study-specific, and not in the $\mathop {\mathtt {MedDRA}}\limits$ terms. For example, by removing the phrase “Baseline Phase”, the $\mathop {\mathtt {AE}}\limits$ term “Throat tightness - Baseline Phase” can be normalized to the $\mathop {\mathtt {MedDRA}}\limits$ term “Throat tightness”. The phrase “Baseline Phase” is given to specify the initial phase of assessment involving collection of initial data in the study, and thus unrelated to the adverse event itself.
- We removed the stop words and lemmatized $\mathop {\mathtt {AE}}\limits$ terms using the NLTK library^31^, and Stanza NLP Library^32^, respectively.
- We mapped an $\mathop {\mathtt {AE}}\limits$ term to its most similar $\mathop {\mathtt {MedDRA}}\limits$ term if their edit distance is less than 4. For example, the adverse event term “Cholecyctitis” will be normalized to the $\mathop {\mathtt {MedDRA}}\limits$ term “Cholecystitis”. This process can correct simple misspellings.

After each step above, if the normalized $\mathop {\mathtt {AE}}\limits$ term is a $\mathop {\mathtt {MedDRA}}\limits$ term, we will stop the normalization. With the above normalization, we successfully normalized 7,296 $\mathop {\mathtt {AE}}\limits$ terms to $\mathop {\mathtt {MedDRA}}\limits$ terms. In total, we got 15,976 unique $\mathop {\mathtt {MedDRA}}\limits$ terms and had 7,393 $\mathop {\mathtt {AE}}\limits$ terms that cannot be normalized.

In order to construct a one-to-one mapping between the adverse events and the $\mathop {\mathtt {MedDRA}}\limits$ terms, we used the $\mathop {\mathtt {MedDRA}}\limits$ dictionary to further group multiple $\mathop {\mathtt {MedDRA}}\limits$ terms of the same adverse event into a unique $\mathop {\mathtt {MedDRA}}\limits$ term. According to the definition of $\mathop {\mathtt {MedDRA}}\limits$, each $\mathop {\mathtt {MedDRA}}\limits$ term is assigned to one of the five hierarchical levels^33^. Specifically, the $\mathop {\mathtt {MedDRA}}\limits$ terms with the lowest level (i.e., level 1), which are used to communicate the adverse events in practice, could correspond to the same adverse event. For example, “Eye itching” and “Ocular itching” are two $\mathop {\mathtt {MedDRA}}\limits$ terms with level 1 and represent the same event. Such $\mathop {\mathtt {MedDRA}}\limits$ terms corresponding to the same event have a common parent, which is a $\mathop {\mathtt {MedDRA}}\limits$ term with level 2 (e.g., “itchy eyes” in the above example). Therefore, we normalized each $\mathop {\mathtt {MedDRA}}\limits$ term with level 1 to its linked $\mathop {\mathtt {MedDRA}}\limits$ term with level 2. In total, we converted 15,976 $\mathop {\mathtt {MedDRA}}\limits$ terms into 11,153 more abstract $\mathop {\mathtt {MedDRA}}\limits$ terms. Each term among these 11,153 $\mathop {\mathtt {MedDRA}}\limits$ terms and 7,393 non-$\mathop {\mathtt {MedDRA}}\limits$ terms represents an adverse event, which is further represented as an *adverse-event* node in $\mathop {\mathtt {CTKG}}\limits$. Note that due to the licensing restriction of $\mathop {\mathtt {MedDRA}}\limits$ $\circledR$, we didn’t specify which *adverse-event* nodes represent $\mathop {\mathtt {MedDRA}}\limits$ terms in $\mathop {\mathtt {CTKG}}\limits$ and only kept the terms as the attribute of *adverse-event* nodes.

### Drug mentions and normalization

In AACT, the drugs used in studies (i.e., clinical trials) could be found in the intervention table, in which the “name” field stores the information about medicines and administrations used in each intervention. For example, we could find that the drug Naltrexone is used in the study NCT04322526 via its intervention “Naltrexone 50 Mg Oral Tablet.”

In $\mathop {\mathtt {CTKG}}\limits$, we used Medical Text Indexer (MTI)^34^ to automatically extract drug mentions. MTI is developed by the National Library of Medicine (NLM) to recognize medical entities (e.g., anatomy, drugs and conditions) from plain text. We used this tool to extract drug mentions following 2 steps:

- We used MTI to automatically recognize all the medical entities from the interventions.
- We found drug entities from the medical entities recognized by MTI. Specifically, for each recognized entity, MTI will output its MeSH code if available. MeSH is a hierarchically-organized vocabulary from NLM to index and categorize biomedical and health-related information^35^. Given the MeSH code, we first identified entities with MeSH codes starting with character “D”, which indicates drug entities (e.g., D02.241.223.701.430 for Ibuprofen). After that we removed the entities not representing specific drugs by excluding those with the MeSH code D26.310 (drug combination), D26 (pharmaceutical preparations), D23.101 (biomarkers) and D26.255 (dosage forms). We also noticed that a few recognized entities were not associated with MeSH codes. For these entities, we did a manual check and identified the ones representing specific drugs.

After the above 2 steps, there were still 1,775 unique interventions in which MTI did not find any drug mentions. For these interventions, we did a manual search and identified the drugs mentioned. Eventually, from the intervention table, we found 3,487 mentioned drugs in total. Among these drugs, 860 (24.7%) of them are found manually. Most of the manually found drugs are investigational drugs (e.g., pf-06669571), or drugs mentioned in abbreviations (e.g., tvr and umec).

Besides the drugs in interventions, there were also drugs mentioned in the titles or descriptions of the study groups (e.g., event group). For example, from the title “tramadol/diclofenac 25/25”, we could find the drugs Tramadol and Diclofenac. We also extracted drugs mentioned in the titles or descriptions of study groups to generate a complete list of drug mentions. Specifically, we first used the above 2 steps to automatically extract the mentioned drugs in titles and descriptions of study groups. For groups that we did not find any drugs automatically, we manually searched their titles and descriptions, and identified the mentioned drugs. In the end, we found 4585 drug mentions from the interventions and the study groups.

From the drug mentions, we observed that one drug could be represented by different names. For example, the drug “losartan potassium” could be represented by its brand name “cozaar” or its generic name “losartan.” Therefore, we normalized the drug mentions found in texts to normalized terms. Specifically, we first used MTI to map all the 4585 drugs to their MeSH terms. For example, MTI could automatically map the drugs “losartan potassium”, “cozaar” and “losartan” to the MeSH term “losartan.” For the drugs that MTI can find their MeSH terms, the MeSH terms were used as their normalized terms. For the other drugs, if they are in abbreviations (e.g., tvr), we first found their full names (e.g., Telaprevir), and used the MeSH terms of their full names for normalization; if they are not in abbreviations, we used their generic names for normalization. We noticed that investigational drugs may not have generic names. For these drugs, their identifiers mentioned in studies (e.g., pf-06669571) were used as their normalized terms. After the normalization, the 4585 drug mentions were normalized to 2548 normalized terms. Each of the normalized term is represented as a *drug-term* node in $\mathop {\mathtt {CTKG}}\limits$.

### Statistical analysis method normalization

We observed that one statistical analysis method could be represented by different names in the table. For example, the method “paired *t*-test” could be represented as “paired *t* test”, “paired *t*-tests” and “paited *t*-test” in the table. Therefore, we normalized the names of the methods using the 3 steps as follows:

- We preprocessed the method names from the table by removing the space and punctuation in the text.
- We calculated the edit distance among the preprocessed names, and normalized the preprocessed names with edit distance less than 4 to a same normalized term. We also did a manual check to correct possible mis-normalization. For the names that will be normalized to a same term, we used the names with the highest frequency as the normalized term.
- We further refined the normalized terms by merging the terms with the same words. We noticed that after the second step, there were still normalized terms that represent the same method with the same words but of different orders. For example, the normalized terms “pairedttest” and “ttestpaired” represent the same method “paired *t*-test” with the same words but of different orders. We manually merged such terms to the one with the highest frequency.

After all the steps, we normalized the 1,299 unique method names mentioned in the table to 907 normalized terms. Each of the normalized terms is represented as a *method* node in $\mathop {\mathtt {CTKG}}\limits$.

### Outcome extraction and outcome clustering

In AACT, the outcome measures used to test the effectiveness of the interventions could be found in the “title” or the “description” fields of the outcome table. Most of the titles in the outcome table are long phrases and could involve multiple standard outcome measures (e.g., in the title “Change From Baseline in Platelet Count and White Blood Cell Count”, where “Platelet Count” and “White Blood Cell Count” represent standard outcome measures). These standard outcome measures are common assessment tools that are used to assess the effectiveness of an intervention. The complex relations between the outcome titles and the standard outcome measures make it difficult to directly represent the outcomes with the extracted standard outcome measures. Therefore, we incorporated the identified standard outcome measures as nodes into $\mathop {\mathtt {CTKG}}\limits$ and built connections between the *outcome* nodes and the involved *standard-outcome* nodes. Through such connections, we can infer which standard outcome measures are used in each study to assess the efficacy of interventions. We observed that some popular phrases within the titles or the descriptions of outcome records represent standardized assessment tools used to measure the outcome of clinical trials, for example, “Visual Analogue Scale” is a tool widely used as a measure for pain. Incorporating such standard outcome measures into the $\mathop {\mathtt {CTKG}}\limits$ could enable the comparison on the outcome measurements across different studies, and also could provide a reference regarding the choice of standardized assessment tools in the design of clinical trials. Therefore, we extracted the phrases that could represent standard outcome measures as below:

- We found the abbreviations and identified the definitions of abbreviations from the titles or the descriptions of the outcomes using the Schwartz-Hearst algorithm^36^. We observed in the titles that many standard outcome measures are associated with their corresponding abbreviations. For example, we could identify the abbreviation “BI” and the corresponding definitions “Bleeding Index” from the outcome name “Gingival Health Measured by Bleeding Index (BI).”
- We kept only the definitions containing the following words: scale, index, score, test, questionnaire, value, count, inventory, assessment, level, rate. We observed that most standard outcome measures would contain such words (e.g., “Visual Analogue Scale”, “Social Responsiveness Scale”).
- We manually normalized different variants of the same standard outcome measures and removed the extracted phrases that are not outcome measures. We also manually added some popular standard outcome measures (e.g., “Overall Survival”, “blood pressure”, “triglyceride”) that do not contain the above words or do not have any abbreviations.

All the extracted phrases are represented as the *standard-outcome* nodes in the $\mathop {\mathtt {CTKG}}\limits$. In the end, we got 492 *standard-outcome* nodes from 50,342 outcome records (i.e., 56.96% over all the 88,386 outcome records), and connected the *standard-outcome* nodes with the corresponding *outcome* nodes.

With the extracted standard *outcome* measures, there were still more than 40% of the outcome nodes not connected to any *standard-outcome* nodes. Therefore, to aggregate similar outcome nodes, we also grouped all the outcome titles (including those containing the standard outcome measures) into several clusters. Specifically, we represented each outcome title using its term frequency-inverse document frequency (TF-IDF) vectors. We then grouped the TF-IDF vectors of outcome titles using the CLUTO^37^, a clustering toolkit, into 200 clusters. Each cluster is presented as a *cluster-outcome* node and has attributes describing the cluster size, that is, the number of outcomes within the cluster, and the most representative words of these outcomes. Specifically, for each cluster, the representative words of outcomes include 5 descriptive words and 5 discriminating words derived by CLUTO that can best describe or discriminate each cluster. Each word is associated with a percentage computed by CLUTO (details in its manual) which indicates the importance of this word with respect to describing or discriminating the cluster. We converted the descriptive words and the discriminating words as two attributes of each cluster, by combining the words and their corresponding percentages. For example, after clustering, one *cluster-outcome* node has these descriptive features: “circumference 56.4%, waist 43.0%, head 0.1%, abdominal 0.1%, change 0.1%”, and most *outcome* nodes connected with it have the titles related to “circumference” and “waist”, such as “Change in Waist-to-hip Ratio” and “Mean Change From Baseline in Waist Circumference”.

## Data Availability

The $\mathop {\mathtt {CTKG}}\limits$ dataset and the code for the embedding analyses are released in GitHub: https://github.com/ninglab/CTKG.
